# Biodegradation of Pig Manure by the Housefly, *Musca domestica*: A Viable Ecological Strategy for Pig Manure Management

**DOI:** 10.1371/journal.pone.0032798

**Published:** 2012-03-14

**Authors:** Helena Čičková, Berta Pastor, Milan Kozánek, Anabel Martínez-Sánchez, Santos Rojo, Peter Takáč

**Affiliations:** 1 Institute of Zoology, Slovak Academy of Sciences, Bratislava, Slovakia; 2 Scientica, s. r. o., Bratislava, Slovakia; 3 Departmento Ciencias Ambientales y Recursos Naturales/Instituto de la Biodiversidad CIBIO, Universidad de Alicante, Alicante, Spain; University of Kentucky, United States of America

## Abstract

The technology for biodegradation of pig manure by using houseflies in a pilot plant capable of processing 500–700 kg of pig manure per week is described. A single adult cage loaded with 25,000 pupae produced 177.7±32.0 ml of eggs in a 15-day egg-collection period. With an inoculation ratio of 0.4–1.0 ml eggs/kg of manure, the amount of eggs produced by a single cage can suffice for the biodegradation of 178–444 kg of manure. Larval development varied among four different types of pig manure (centrifuged slurry, fresh manure, manure with sawdust, manure without sawdust). Larval survival ranged from 46.9±2.1%, in manure without sawdust, to 76.8±11.9% in centrifuged slurry. Larval development took 6–11 days, depending on the manure type. Processing of 1 kg of wet manure produced 43.9–74.3 g of housefly pupae and the weight of the residue after biodegradation decreased to 0.18–0.65 kg, with marked differences among manure types. Recommendations for the operation of industrial-scale biodegradation facilities are presented and discussed.

## Introduction

The production and disposal of large quantities of agricultural waste is a recurrent problem in many countries throughout the world. In pig farms especially, the problem is exacerbated by the concentration of these facilities in small areas, exceeding the assimilation capacity of the environment. The common method of manure management in small to medium farms is to apply livestock waste to crop and forest surfaces that are close to the farms, after storing it for several months in lagoons [1]. The great amount of waste applied to the soil contributes to environmental pollution, due to the presence of pathogens and the leaching of excess nitrogen, phosphorus and other elements, which may contaminate soil and water [2]–[5]. Several technologies have been proposed for recycling wastes from farms: liquid and solid fractions separation, aerobic or anaerobic digestion, composting, fermentation, lagooning, etc. [6], [7]. However, all of these alternatives present associated problems, either because their cost is relatively high, or because of the production of toxic and polluting substances. The majority of farms in the regions of study (Slovakia and Spain) are small or medium scale; farms are mainly family facilities with economic limitations that inhibit them from applying current recommended manure management technologies [1]. There is a need to find new and affordable technologies that solve this environmental problem.

Dipterans and other coprophagous insects are important in nature due to the fact that they degrade organic matter from faeces and transform it into biomass [8]. The residual organic matter, which has not been assimilated, is also decomposed and used easily by plants and other organisms [9], [10]. When compared with other invertebrates, insects are, potentially, more active agents for biodegradation due to the fact that their developmental periods are relatively short. Larvae of many species of dipterans are especially interesting as they are able to develop in a wide diversity of media, have a high reproductive capacity and a relatively short life cycle [11]. For some years, the degrading potential of some groups of invertebrates has been evaluated in terms of their potential to recycle manure produced by livestock. The groups used with success thus far include earthworms (vermicompost) and larvae of dipterans [12]–[15]. However, most of these assays have been done on a small scale. Given that pig manure production can reach 41 million metric tons per year, as in Spain [16], a technology with industrial dimensions has to be designed in order to assimilate these quantities of waste. Although Diptera species with coprophagous larvae have been considered traditionally as pests [17], assays in the United States proved that using some dipterans is an economically viable alternative for degrading livestock byproducts on farms [14]. Degradation by feeding fly larvae reduces the water content, odor and nitrogen levels in manure and leaves less waste material for disposal, compared with undegraded manure [18]. Previous research has used *Musca domestica* (Linnaeus, 1758) (Diptera: Muscidae) for recycling manure [13], [18], [19], as it is the most frequently found species in animal manure, has non-specific rearing requirements and a relatively short lifecycle. Another suitable species for biodegradation, the black soldier fly, *Hermetia illucens* (Linnaeus, 1758) requires a much warmer environment, with most of the oviposition occurring at 27.5–37.5°C [20]. Managing high temperatures in temperate regions might prove difficult and energy consuming. Additionally, the developmental period of the black soldier fly under optimum conditions is much longer. The black soldier fly larval development can take from 10–31 days up to 4 months and the pupal stage usually lasts for another 2 weeks, with these times depending on temperatures and the quality and quantity of the larval medium used [21]–[23].

The great number of flies needed to catabolize a reasonable amount of manure and the large space needed for biodegradation still pose problems [7], [24], [25]. Rearing of large numbers of houseflies has been limited to artificial media and the use of resulting pupae as hosts for hymenopteran parasitoids [26]. While many aspects of house fly ecology have been well studied, mass rearing of the housefly still involves many areas which have not been sufficiently surveyed and impede the release of the technology for industrial production. As a result of the EU LIFE project ECODIPTERA, two pilot plants were built to develop the necessary technology to process manure produced by a pig farm and to evaluate the suitability of different types of pig manure for larval development and biodegradation. One pilot plant, which processed fresh pig manure, was built in Miloslavov (Slovakia); the other plant for processing of pre-treated (centrifuged) slurry was located in Alpuente (Spain). In the present paper, we aim to describe the technology applied to the treatment of swine manure with housefly larvae and the process required to assimilate large volumes of livestock waste. Both facilities: their structure, maintenance, rearing methods of adults and larvae, optimization of the process and assessment of the efficiency of biodegradation are presented.

## Methods

### Rearing of adult flies

**Figure 1:**
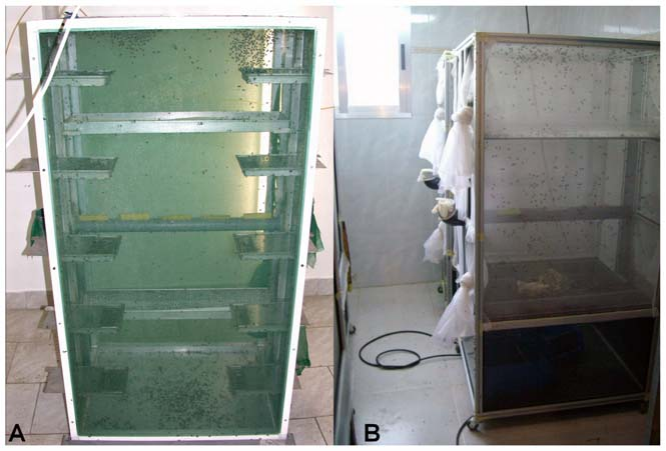
Production cages used to rear adult houseflies. (A) Miloslavov cages, (B) Alpuente cages.

In the Miloslavov pilot plant the housefly colony was established in 2005 by combining adults from an existing laboratory strain of houseflies with adults that emerged from pupae found in manure from the pig farm in Miloslavov (Slovakia), as previously described [27]. Adult flies were kept at 25±2°C with a photoperiod of 12∶12 (L∶D) and relative humidity of 45–60%. In the larval rearing room, conditions were kept at 24±2°C, 12∶12 (L∶D) and ambient air humidity. Flies were maintained in two types of cages: experimental cages (30×30×30 cm) at medium densities (1,000–1,500 pupae per cage) and production cages (60 cm long, 80 cm wide, 145 cm high; Fig. 1A) designed within the project, which could be loaded with up to 25,000 pupae and were used primarily for egg production. Thus, the volume available for the adults in production cages under these conditions was 2.83 cm^3^ and the area available was 2.80 cm^2^ per fly. The production cage consisted of two walls of fine gauze (0.2 mm mesh size) covering the wide sides of the cage and two narrow walls made of stainless steel with two gauze sleeves to allow manipulation of the contents of the cage. A U-shaped plastic tube (5 cm diameter) is placed in the middle of the cage through the narrow walls and filled with water. Five longitudinal apertures in the tube are used to insert sponges, which soak up the water and serve as drinking sites. Afterwards, two aluminum trays (4×58×16.5 cm) are placed inside the cage: the upper one with food (a mixture of powdered milk and sugar in a 1∶1 ratio) and the lower one with pupae 2–3 days before expected emergence. Each narrow metal wall features five drawers (13.5 cm long, 18.5 cm wide, 3 cm high), which can be used for egg collection. Cages are provided with food and water *ad libitum*. Starting from day 5 after emergence, the flies are provided with oviposition substrate (fresh pig manure) offered in special oviposition devices [27] and placed at the bottom of the cage. Flies are allowed to oviposit for 12–14 hours daily for a period of 15 days. After egg production, the food and water is removed from the cages and the flies are left to die. The cages are then disinfected and prepared for the next rearing cycle. To evaluate the effectiveness of rearing the flies in this manner, three production cages were set up with 25,000 pupae in each one and the volume of eggs collected during the egg production period was recorded (1 ml≈11,000 eggs).

In the Alpuente facility, the houseflies were obtained from colonies which had been maintained in the laboratories of the University of Alicante since 2006. Three different strains were used: Slovak (2006, laboratory strain), Spanish (2006, urban strain) and Venezuelan (2007, necrophagous strain). Environmental conditions in the adult rearing room were the same as in the Miloslavov facility. One large production cage (70 cm long, 80 cm wide, 150 cm high; Fig. 1B) was loaded with 40,000 pupae for each strain, except the Slovak strain, which was replicated twice. These cages have two walls of gauze covering the wide sides and two narrow walls made of dark plastic, with gauze sleeves provided to allow manipulation of the contents of the cage. As in the Miloslavov model, a U-shaped plastic tube is placed in the middle of the cage through the narrow walls and filled with water, with three longitudinal apertures where sponges are inserted to facilitate extraction of water by adult flies. Two plastic trays are placed above the tube: one of them with pupae from which adult flies will emerge and the other one with food (sugar and milk powder in a proportion of 2∶1). For egg collection, three boxes (22 cm long, 33 cm wide, 16 cm high) containing one small tray (8.5 cm long, 19 cm wide, 4.5 cm high) each, containing oviposition medium (pig manure), were placed in the bottom. Oviposition substrate was offered since the first day after emergence for 17 hours every day for a period of 5 weeks. In each week, there was a period of 3 days when oviposition substrate was not offered to adult flies. Eggs were collected and measured volumetrically. After this period of 5 weeks, food and water was removed from the cages, the flies were eliminated with a vacuum cleaner, and cages were cleaned and prepared for the next cycle.

### Biodegradation of manure

Pig manure used in the experiments described in this paper was obtained from pigs of three origins: two commercial pig farms (Miloslavov and Alpuente), and from CTA-IVIA (Centre of Animal Technology; Alpuente). The pigs were, at the time of this study, reared for commercial purpose (meat production) and thus ethics approval was not required.

**Figure 2:**
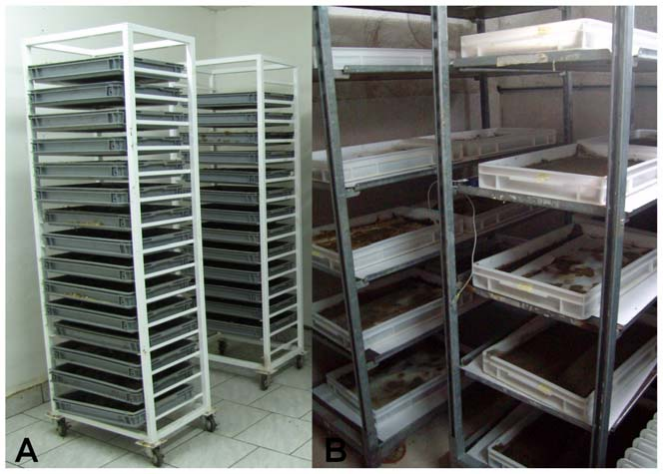
Trolleys with larval trays for biodegradation. (A) Miloslavov trolleys (maximum capacity 75 kg of manure) and (B) Alpuente trolleys (maximum capacity 64 kg of manure).

In the Miloslavov pilot plant the manure was manually collected from the pig pens on a daily basis. Two types of fresh pig manure were used. The first type of manure came from the lactating sows and unweaned piglets. This manure contained variable amounts of sawdust (∼50% of volume), which was used as bedding for the pigs. The moisture ranged from 70 to 80%. The second type of manure was obtained from pens with weaned pigs fed by a standard growing diet and contained no sawdust. The moisture ranged from 65 to 85%. The manure was often nearly semi-liquid. Once the manure was collected, it was transported to the facility and loaded into larval rearing trays (shallow plastic containers; inner dimensions: 37 cm long, 47 cm wide, 7 cm high). The holding capacity of each tray was 5 kg of manure. The manure was weighed and spread into trays. The exact volume of eggs (0.4 ml of eggs/kg of manure with sawdust and 1 ml of eggs/kg of manure without sawdust) prepared in a calibrated tube was seeded on top of the manure with 2–5 ml of water. After egg seeding, trays were placed in trolleys (46 cm long, 73 cm wide, 190 cm high; 15 trays per trolley; Fig. 2A) and kept in the larval rearing room until the biodegradation was completed.

For evaluation of larval development in the two types of manure (manure with sawdust and manure without sawdust), freshly collected manure was packed into plastic sacks and frozen at −20°C for 3–4 days to kill other organisms present therein. The manure was left to warm to room temperature 24 hours before the start of the experiment. The required amount of manure (5 kg) was weighed, spread into larval trays, and seeded with an appropriate amount of housefly eggs. Prior to egg seeding, a sample of 100–150 eggs was taken directly from the tube with the calibrated amount of eggs and placed on a piece of moist sponge cloth in a Petri dish. The hatchability of eggs was evaluated by counting the number of hatched and non-hatched eggs after 24 hours of incubation at 25±2°C, 45–60% RH and 12∶12 h L∶D. The percentage of egg hatch was used to estimate the initial number of house fly larvae. Larval trays with seeded manure were kept in the larval rearing room until the larvae pupated and then the pupae were recovered from the manure residue by flotation in water. The percentage of larval survival was estimated as the number of pupae recovered from the larval tray, divided by the initial number of larvae in the manure for the respective larval tray (based on egg hatchability) and multiplied by 100. Five hundred air-dried pupae were weighed (±0.0001 g) to check the mean weight of pupae, which was used to estimate the total weight of biomass recovered from manure. The weight of manure residue after biodegradation was calculated as the weight of manure with pupae at the end of biodegradation minus the weight of biomass recovered by flotation in water. Twenty replicates were evaluated for each manure type and egg hatchability, larval survival, mean weight of pupae, total weight of biomass and weight of manure residue were calculated individually for each replicate.

In the Alpuente facility, manure used was also manually collected from two different origins. One type was collected in CTA-IVIA (Centre of Animal Technology). This manure was obtained from experimental farms in the form of slurry and then dehydrated by a method of solid-liquid fractions separation with a decanter centrifuge (73% of moisture content). The second type of manure, with 80–85% moisture content, was obtained directly from the pens of the farm, to avoid any anaerobic process that could affect the manure during storage. The first kind of manure was called centrifuged slurry and the second was called fresh manure. Once manure was collected, it was transported to the pilot plant and stored in a freezer at −20°C to kill any invading arthropods that could have developed in it. One day before beginning the experiments, closed pots with manure were allowed to warm to room temperature; after defrosting, 4 kg of manure was placed in rearing trays (40 cm long, 60 cm wide, 7.5 cm high). Once trays were set with pig manure they were seeded with eggs (0.5 of eggs/kg of centrifuged slurry and 0.8 ml of eggs/kg of fresh manure) following the same method used in the Miloslavov pilot plant. Trays were placed in trolleys (56.5 cm long, 135 cm wide, 189 cm high; 16 trays per trolley; Fig. 2B) and left in the larval room under similar conditions as in the Slovak facility; only photoperiod was different because, in this pilot plant, natural light is provided. In total, fifteen replicates were set, trays were left in the biodegradation room and from the fifth day of larval development they were weighed daily, until day 10, when pupae were removed from the medium. Then, pupae and manure residue were weighed separately. Total weight of pupae divided by the mean weight of one pupa was the estimated number of pupae that survived the larval stage.

### Processing of final products

In the Miloslavov facility, with the exception of a small percentage of processed manure subjected to floatation in water, to recover pupae used to maintain the egg production colony, the degraded manure with pupae was loaded into plastic sacks and placed in a freezer for 4 days at −20°C to kill the pupae present therein. Once the pupae were killed, the processed manure was left to air-dry on the trays or screens, then milled and packed. Since freezing as the means of killing the pupae left in manure is lengthy, expensive, and limited by the capacity of the freezer, the possibility of employing a microwave oven (DIES 3V, Czech republic) for drying was evaluated. Oven size allowed a 4 larval tray capacity. Two drying regimes of different intensity and duration were tested. The moisture content of manure samples taken from each larval tray, before and after the selected drying regime, was evaluated by drying the samples at 40°C for 24 hours. This temperature was chosen because the samples tended to self-combust in a warmer environment (>60°C).

In the Alpuente facility, after 10 days of larval processing, pupae were separated from the degraded manure with sieves of different sizes. The first sieve (2.5 mm) separated large pupae from coarse pieces of manure and the second sieve (1.5 mm) separated small pupae from fine, degraded manure. Degraded manure fertilizer and pupae were kept in the freezer, in the case of pupae to avoid emergence of adult flies and in the case of fertilizer to maintain its nutritional properties.

### Structure and maintenance of the facility

**Figure 3:**
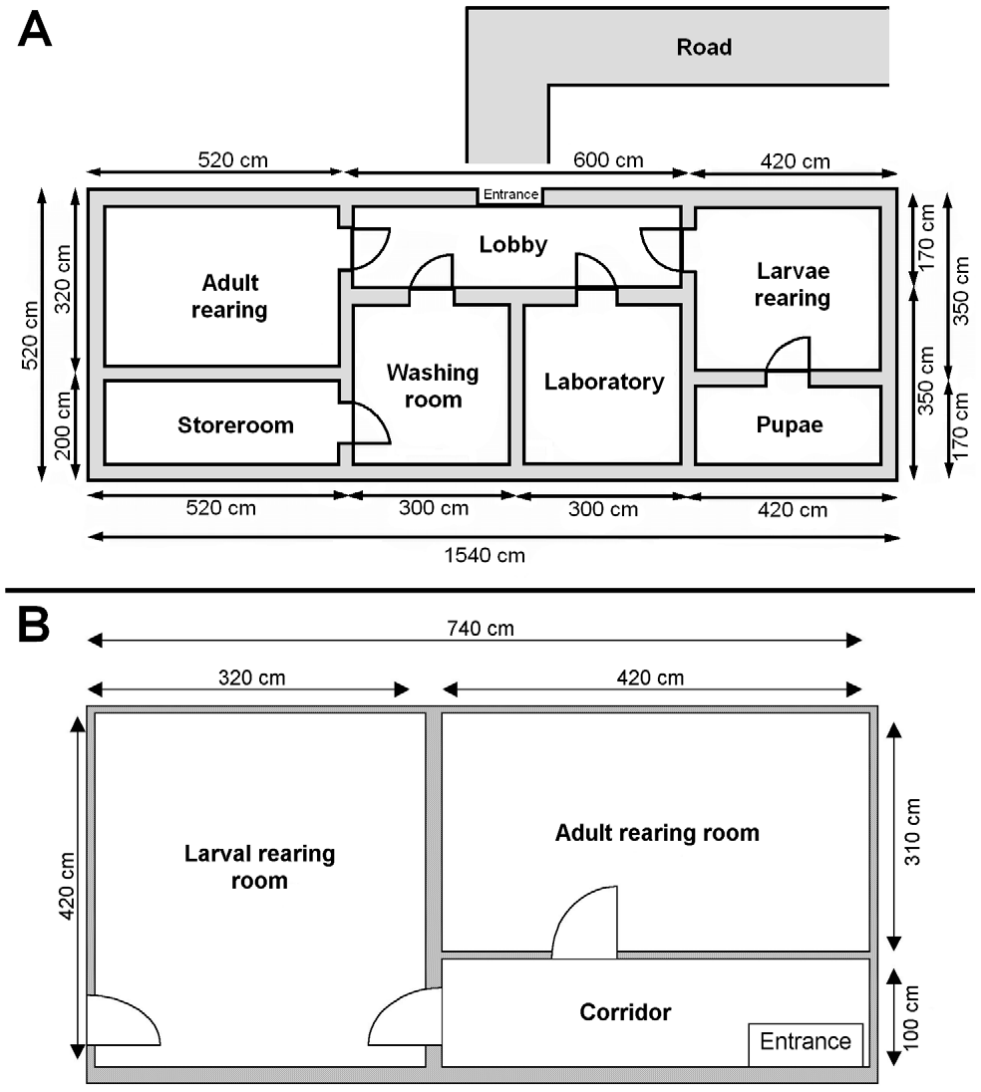
Floor plans of the biodegradation facilities. (A) Miloslavov pilot plant, (B) Alpuente facility.

The Miloslavov experimental pilot plant was located within the area of a pig farm. The key elements of the facility include the adult room, where the egg-production colony flies are kept, and the larval rearing room, where the manure biodegradation process takes place. In addition, the facility includes a washing/work room (for egg collection, handling of manure, and cleaning procedures), a drying room (for drying processed manure, from which the larvae/pupae were removed or killed), a storage room (for keeping the products of biodegradation), a laboratory (for running experiments), a toilet, and a lobby connecting these rooms (Fig. 3A). The facility and all equipment were cleaned on a regular basis with commercial sodium hypochlorite-based disinfectant. Equipment and surfaces coming into direct contact with manure and/or flies (i. e. rearing trays, oviposition cloths, screens, cages, shovels, etc.) were disinfected immediately after use. Prevention of fly escape was ensured by sticky tapes placed in both egg production and biodegradation rooms and by a UV trap in the biodegradation room.

The Alpuente pilot plant was built in 2008 in Alpuente, a village located in the Valencia region, Spain. The facility was attached to a closed-cycle farm, from which pig manure could be obtained for processing in the pilot plant. This building was previously used as a farm laboratory, where semen for insemination of female pigs was prepared. It consisted of two rooms separated by a corridor. The first room was used as the adult room, where colonies of adult flies are reared, and the second room as the larval rearing room, where the biodegradation process occurs (Fig. 3B). Washing and working areas were included in the adult room and a freezer in the larval room.

### Data analysis

Due to marked differences in housefly strains, rearing techniques and methodology of the experiments, data presented in this study were not compared statistically. Descriptive statistics were calculated using the data analysis tool of MS Excel.

## Results and Discussion

### Rearing of the adult flies

**Figure 4:**
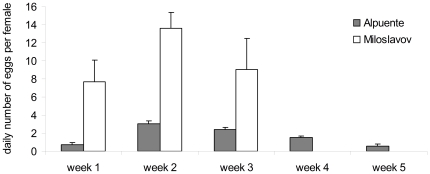
Productivity of cages with adult houseflies. Daily number of eggs per female (mean ± SE) collected in adult cages during the first 3 weeks in the Miloslavov facility (15 days, from the 5^th^ day after emergence) and during 5 weeks in the Alpuente facility (31 days, from the 1^st^ days after emergence). Miloslavov cages contained 25,000 pupae (n = 3). Alpuente cages contained 40,000 pupae (n = 4).

Although the design of the production cages was similar at both facilities, better results were achieved in the Miloslavov pilot plant (Table 1). Flies laid the largest quantity of eggs during the second week after emergence in the Miloslavov pilot plant. However, if eggs were collected from the end of the first week after emergence, a relatively stable amount of eggs could be collected during the following weeks (week 2 and 3), with a slight decrease during the third week. Similar results were observed in the Alpuente cages, but with lower overall productivity (Fig. 4). Larger quantities of eggs were collected during the second week and quantities gradually decreased during the 3^rd^, 4^th^ and 5^th^ weeks. These results suggest that after a period of three weeks following emergence, the flies should be removed/killed, the cage and all its compartments disinfected and prepared for the next oviposition cycle.

**Table 1:** Egg production obtained from the two models of cages during a 15-day egg-collection period.

Cage model	Replicates	Pupae	Total volume of eggs (mean ± SE) produced during sampled period (ml)	Daily egg production (mean ± SE) (ml)	Maximum daily egg production (ml)	Minimum daily egg production (ml)
Miloslavov	3	25,000	177.7±32.00	11.85±2.13	18.70	5.00
Alpuente	4	40,000	44.40±2.93	2.99±0.63	9.60	0.20

A single cage in the Miloslavov pilot plant loaded with 25,000 pupae produced on average 177.7±32.0 ml of eggs (11.85±2.13 ml per day) in a 15-day egg-collection period, while a cage loaded with 40,000 pupae in the Alpuente facility produced on average 44.4 ml of eggs in all sampled periods (2.99±0.63 ml per day) (Table 1). This marked difference in egg production is probably the result of too high a density of flies in the Alpuente cages, resulting in a high mortality rate in the early days after emergence. It has been previously observed that the fecundity of various insects depends on the adult population density [27], [28]. Differences in the fly strain and adult diet could also play an important role, although the number of eggs collected during the oviposition period was lower than previously reported for the same housefly strain under similar environmental conditions and adult population density [27]. This difference probably reflects further negative effects on their fecundity, due to overpopulation of caged houseflies. Moreover, the production cages used in the Alpuente facility differed slightly from the ones used in the Miloslavov facility and some differences, such as the egg collection method or other aspects of the design, could have negatively affected the egg production in the Alpuente facility.

### Biodegradation of manure

The four types of pig manure that were tested proved to have different properties and suitability for biodegradation (Table 2). In the Miloslavov facility, long-time empirical observations showed that the optimal quantity of housefly eggs for successful biodegradation is 0.4 ml (4,400 eggs)/kg for manure with sawdust and 1 ml (11,000 eggs)/kg for manure without sawdust. This difference was expected; sawdust apparently has no nutritional value for the larvae and from this point of view is more or less redundant. However, despite the negative effects on the nutritional value of manure, the presence of sawdust proved to be beneficial for the development of the larvae in manure. The survival of larvae in manure containing sawdust was relatively high, probably due to its loose structure, good aeration of the substrate or a better C/N ratio [29]; newly-hatched larvae readily buried themselves in this type of manure, as opposed to the manure without sawdust, where the larvae were crawling on the surface during the first days after hatching. In the case of manure without sawdust, larval survival was low due to unfavorable conditions (lack of oxygen, excessive moisture, semi-liquid consistency) and ranged between 35–50%. Additionally, development of larvae and biodegradation was faster in manure with sawdust. Generally, the larvae pupated on the 6th–7th day following seeding in manure with sawdust and on 9th–10th day in manure without sawdust (Table 2). In the case of manure without sawdust, larval development and biodegradation were slower (3–5 days longer than in manure with sawdust). The likelihood of anaerobic processes in this type of pig manure was also supported by the presence of some dipteran larvae (*Eristalis* sp.) in the manure without sawdust, which are common in anaerobic substrates. Limited suitability of manure under anaerobic conditions for biodegradation by housefly larvae was also noted by [30], who reported the necessity of aeration or further modification of such substrate to support larval growth.

**Table 2:** Larval development (mean ± SE) in different types of manure.

Manure type	Replicates	Maximal larval period^a^ (days)	Number of harvested pupae^b^	Larval survival (%)	Total weight of pupae^b^ (g)	Weight of manure residue^b^ (g)
Manure with sawdust	20	7	2,886.34±132.93	73.302±3.13	43.85±2.06	650±0.01
Manure without sawdust	20	11	4,653.68±191.78	46.870±2.143	74.29±1.67	270±0.01
Centrifuged slurry	15	8	2,778.43±415.79	76.78±11.87	-	360±0.01
Fresh manure	15	9	3,551.65±465.11	61.34±8.30	-	180±0.01

a the number of days from egg seeding day until most of the larvae have pupated.

b the values are expressed per 1 kg of manure.

In the Alpuente facility, both fresh manure and centrifuged slurry showed faster biodegration than the manure without sawdust. Among all tested manures, larval production was best in centrifuged slurry, in terms of both survival and speed of development (Table 2). As noted, the water content of manure slurry obtained in intensive farms is high (around 97–98% moisture). As the region surrounding the Alpuente pilot plant is semi-arid, manure management should recover available water. Physical solids separation by auger or centrifuge can produce water suitable for crop application or reuse in farms [31], and the solid fraction can be degraded by housefly larvae. In the decanter centrifuge, a horizontal cylinder rotates continuously at high velocity and the centrifugal force separates the liquid fraction, which is deposited in the outer cover, from the solid fraction, which stays inside the cylinder and is continuously augered out [1]. Solid residue obtained in the process is suitable for housefly degradation, due to the fact that larvae can develop at moisture levels from 50 to 80% but not at 40 or 90% [32]; moisture is optimal (73%) and homogeneity of the manure is suitable for separation of the pupae from degraded manure.

Weight loss of the larval medium during biodegradation varied among the tested types of pig manure. The weight of residue of manure with sawdust was on average 2–3 times higher compared to the other manures (Table 2). This was later determined to be the result of retaining a high moisture level, which often reached 60–70% in degraded manure with sawdust, but dropped to 20–30% in degraded manure without sawdust (H. Č., unpublished data). The weight of fly biomass obtained after successful biodegradation reached 43.85 g/kg of manure with sawdust and 74.30 g/kg of manure without sawdust on a wet basis. When sawdust was absent from the manure, the development was slower, but the total yield of pupae increased by almost 70% compared to the manure with sawdust. The low yield of pupae in the presence of sawdust indicates its apparent low nutritional value for the larvae. Comparison of these results with other studies is limited because most of the other authors examined biodegradation potential of the housefly or black soldier fly larvae reared in poultry manure. Differences in terms of both larval survival and pupal mass were observed for houseflies reared in pig and poultry manure under the same environmental conditions [32]. However, [18] observed a 64.4% decrease in manure mass when the housefly larvae were reared at a density of 300 larvae/100 g poultry manure, an 80.3% decrease in manure mass at a density of 600 larvae/100 g of poultry manure, and a 59.1% decrease at a density of 900 larvae/100 g of poultry manure. The results of our experiments partially match these observations: in the four manure types examined here, we recorded a decrease in manure mass of 64.0% at a density of ≈360 larvae/100 g of centrifuged pig slurry, 82.0% at a density of ≈600 larvae/100 g of fresh manure, and 72.8% at a density of ≈1000 larvae/100 g of pig manure without sawdust. The lowest decrease in manure mass was recorded for the manure with sawdust, where the weight of manure decreased by 35.2% at a density of ≈400 larvae per 100 g of manure and was most likely the result of retaining a high moisture level in the manure residue. The weight of biomass recovered from pig manure in the Miloslavov facility is similar to the yield of housefly larvae reared in poultry manure as reported by [33] (3–4 g/100 g of manure) and, on a wet matter basis, compares favorably with the yield of black soldier fly prepupae (46 g/kg of manure) in a poultry manure management system (based on 56 and 74% moisture content of black soldier fly prepupae and poultry manure, respectively; [14], Sheppard, personal communication). When calculated on a dry matter basis, based on average pupal and manure moistures of 72.7 and 75.0%, respectively (H.Č., unpublished data), the yield of housefly pupae in the Miloslavov pilot plant reached 4.8–8.1%, compared to the 7.8% yield of the black soldier fly prepupae reared in poultry manure [14].

### Processing of final products

Following the separation of larvae and pupae from spent manure, separated larvae can be left to pupate or can be handled immediately. Manure residue after biodegradation can be relatively rich in moisture (15–70%, depending on manure type), and often contains extrinsic particles like swine hair or plant material, which is not biodegradable by the larvae (for example, residues of hulls from the animals' diet, sawdust, etc.). For easier manipulation and storing of the product, additional drying and homogenization (milling/grinding) of processed manure should precede individual packing. Effectiveness of drying in a microwave oven was found to be low; manure moisture decreased only by 12 to 23% (Table 3) and self-combustion was occasionally noticed, possibly as a result of too high temperature during drying. Cost of such drying is high when considering the capacity of the drier, power demands of the oven and time of drying. On the other hand, air-drying at laboratory temperature requires 3–4 days with no additional energy cost. However, it requires additional space and the larvae/pupae left in the manure residue must be killed (for example, by freezing or heat) before placing processed manure on trays and meshes.

**Table 3:** Effectiveness (mean±SE) of using a microwave oven for drying of manure residue after processing by house fly larvae.

Program	Total time of drying (hours)	Energy cost	Total weight of processed manure to be dried (kg)	Initial manure moisture (%)	Decrease in moisture after drying (%)
P2	4.4	4.64 kW·h	10.9	61.155±0.896	−12.308±3.637
P3	5.2	7.76 kW·h	7.6	48.470±10.483	−23.880±7.467

### Structure and maintenance of the facility

**Figure 5:**
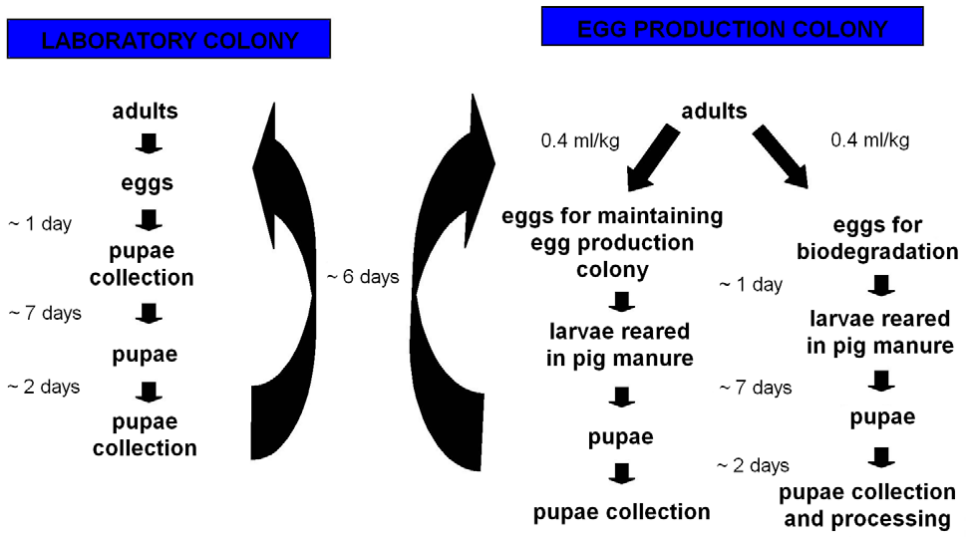
Developmental time of the different stages of houseflies and the different colonies in the facility.

The working scheme of the facility and how colonies should be maintained can be observed in figure 5. The egg production colony was kept in the pilot plant; this colony provided eggs for both the biodegradation process and maintenance of the egg production colony. A laboratory colony was also maintained in a different building in order to prevent any problem that could affect the viability of the production colony; if this happened, the production colony was replaced with flies obtained from the laboratory colony. In Slovakia, the laboratory colony was kept in the insectaries of the Institute of Zoology of the SAS; in Spain, in the laboratories of Institute CIBIO, in the University of Alicante.

The basic space requirement for a biodegradation plant is at least three rooms: adult room, larval (biodegradation) room and a storage room. Large biodegradation facilities should, due to higher demands, also include support areas: a washing/work room (for egg collection, handling of manure, and cleaning procedures), a separation room (with special environment for separating larvae or pupae from processed manure), a drying room (for drying processed manure from which the larvae/pupae were removed or killed), a laboratory (optional, for quality control procedures and other experiments), a room for processing the products (milling, packing), and a social room for staff (including toilets and showers). The support areas can be air-conditioned separately, according to specific environmental needs.

To optimize space in the facility, the area of the adult room (*S_A_*) is calculated from the number of large cages *N_C_* (1) (Table 4), where *i* is the seeding ratio of eggs on manure, *m* is the weight of manure per week (kg) and *p* is egg productivity of a single large cage per week (83 ml). Multiplication by the factor of 2 indicates that for continuous egg production, new cages must be set up with the pupae and the flies must emerge and mature (which takes about a week) before the flies from the previous egg-production cycle can be killed and their cages cleaned. To calculate final space requirements of the room S_A_ (2) a manipulation area must be included, where *s* is size of the base of the large cage and *w* is space for manipulation. Based on the operation of the Miloslavov pilot plant, the manipulation area *w* should be no less than about 150% of *N_C_ · s*.
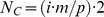
(1)

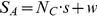
(2)


**Table 4:** Hypothetical number of cages (N_C_), number of trolleys (N_T_), and dimensions of adult (S_A_) and biodegradation (S_B_) rooms for facilities processing 1–10 tonnes of manure per week based on formulas 2 and 4, depending on the type of manure used.

	Number of adult cages^a^: N_C_		Dimensions of adult room: S_A_(m^2^)		Number of trolleys^b^: N_T_		Dimensions of biodegradation room: S_B_(m^2^)	
Manure type	1 tonne	10 tonnes	1 tonne	10 tonnes	1 tonne	10 tonnes	1 tonne	10 tonnes
Manure with sawdust	10	96	12	115.2	14	133	14.1	134
Manure without sawdust	24	240	28.8	288	21	210	21.2	211.6
Fresh manure	20	194	24	232.8	16	153	16.1	154.2
Centrifuged slurry	12	120	14.4	144	18	172	18.2	173.3

a number of adult cages calculated according to formula (1) and based on the egg production of production cages in the Miloslavov pilot plant (83 ml of eggs/week).

b number of trolleys calculated according to formula (3) and based on the holding capacity of trolleys in the Miloslavov pilot plant (75 kg of manure/trolley).

In the case of the larval rearing room, when comparing capacity of the trolleys and trays, space is better optimized in the Miloslavov facility. In the Alpuente plant, in each trolley, 64 kg of manure are degraded, while in the Miloslavov plant, 75 kg. The space requirements for the biodegradation room can be calculated similarly to the dimensions of the adult rearing room (Table 4):
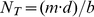
(3)Where *N_T_* is the number of trolleys necessary for biodegradation of a proposed amount of manure per week, *m* is the weight of manure (kg) per week, *d* is the replacement rate (length of larval development (days)/7 days) and *b* is the capacity of the trolley (kg). The space requirement for the biodegradation room (S_B_) will then be:
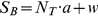
(4)Where *a* is the area occupied by the trolley and *w* is manipulation and working space, which should be no less than about 200% of *N_T_ · a*.

In the adult rearing room, the temperature was set to 25±2°C in both facilities. [34] compared oviposition of adult houseflies at different temperatures (20, 25, 30 and 35°C); flies maintained at 25°C reached the highest mean fecundity of 729 eggs per female (meant as the number of eggs a single female could oviposit during her lifetime), followed by 30°C (707 eggs per female). However, decreased longevity of the flies was observed as the adverse effect of keeping the flies at higher temperature. We suppose that keeping the flies at a temperature of 25–27°C can provide optimal egg yields and maintain sufficient length of the egg-collection period (2 weeks). The humidity in the egg production section should be maintained between 45–60%; higher values increase the risk of fungal infection of adult flies, especially in warm seasons when spores of the fungus are naturally present in the air. High air humidity can also have unfavorable effects on the adult food. Absorption of excessive water vapor can lead to formation of crusts on the surface of food [26] and growth of various saprophagous moulds, which take away the nutrients and can produce toxins.

Temperature is an important factor, which is difficult to regulate in the biodegradation room. At about 25°C, the biodegradation process and larval development are optimal (B.P., unpublished data). Due to different air supply requirements, the egg production section and the biodegradation section should ideally have separate and independent air-handlers. While the egg production section has relatively constant cooling/heating requirements, the biodegradation section often has different demands, because this process generates a significant amount of heat, which affects ambient temperature and varies according to the activity of the larvae and the volume of processed manure. Additionally, a 2–3°C gradient was observed in both adult and biodegradation rooms during the cold season, which can affect the speed of biodegradation and the development of larvae. A single fan installed in the ceiling of both rooms could remove the gradient. Humidity in the biodegradation room should be kept below 60–70% to ensure sufficient drying of the manure during biodegradation, prevent larvae from escaping and avoid their high early mortality. Additional desiccators and frequent air exchanges with air filters might be necessary in large biodegradation rooms, due to the large volume of gases developing during decomposition (ammonia, carbon dioxide, water vapor). The high level of ammonia in the biodegradation room was especially disturbing, because it irritated the eyes and respiratory systems of the staff and limited the length of time they could spend in the biodegradation room. Feasibility of an ammonia trapping system should be evaluated, since it could be a valuable by-product. [35] estimated that, in a biodegradation system using blowfly larvae, as much as 22% of the nitrogen present in pig manure could be “lost” in the form of volatile compounds. We have not observed negative effects of the natural photoperiod on larval development rates; the time needed for the larvae to mature and pupate was similar in both facilities when similar manure types were used.

To maintain a pathogen-free environment inside the facility, all equipment, as well as the floor and walls of each room, were disinfected on a regular basis with commercial sodium hypochlorite-based disinfectant, following manufacturer's instructions. Equipment and surfaces that came into direct contact with manure and/or flies (i. e. rearing trays, oviposition cloths, screens, cages, trowels, etc.) were disinfected immediately after use. During 3 years of operation of the pilot plant in Miloslavov, the adult colony twice contracted fungal infection. Both infections occurred during extremely rainy weather and were connected with incontrollable high humidity of the air in the adult rearing room (over 80%). All the adults were killed at the first sight of the disease, the adult room and all its equipment were thoroughly disinfected and the colony was re-established from fresh stock pupae. In the Alpuente facility, one infection by the fungus *Entomophtora infestans* also took place under the same environmental conditions as in the Miloslavov facility. Colonies were killed and material disinfected.

### Recommendations

Quality control tests of the process should be carried out once a month. These tests should validate the quality of the rearing process (quality of individuals: emergence tests, pupae weight tests) and quality of the biodegradation process (chemical and microbiological tests of the final product).

The following staff should be employed in the facility: 1) one part-time post-graduate, for overall process control, planning and supervising the technicians' duties, development of process improvements, and solving the problems that may arise in the facility; 2) full time technicians (the number depends on the size of the facility) for maintaining adult colonies (in the laboratory and the biodegradation pilot plant), collecting the eggs, collecting manure, preparation of trays with manure and seeding manure with eggs, separation of final products (pupae and manure), preparation and development of quality control tests, cleaning, and record keeping. Shift work would be required.

### Conclusions

Biodegradation of swine manure by housefly larvae is a viable and ecological strategy for pig manure management and compares favorably with a similar method of biological processing of animal waste, which employs black soldier flies. The number of eggs obtained from production cages loaded with 25,000 pupae is sufficient for processing of large quantities of pig manure (ca. 178–444 kg of manure per 1 cage in 2 weeks). Design of production cages can be optimized according to the preferred method of egg collection. Our results show that the optimal amount of housefly eggs needed for biodegradation of different types of pig manure, as well as the weight of acquired fly biomass and manure residue can vary considerably. Thus, before commencing the operation of any large-scale biodegradation plant, we recommend examination of the nutritional value of the substrate that will be processed by the larvae and its suitability for larval development (larval survival). This will help to determine the sufficient number of eggs necessary for biodegradation of the available amount of waste and the most accurate space requirements of the key areas of the biodegradation facility (adult and larval rooms).
